# Trajectory of post-COVID brain fog, memory loss, and concentration loss in previously hospitalized COVID-19 survivors: the LONG-COVID-EXP multicenter study

**DOI:** 10.3389/fnhum.2023.1259660

**Published:** 2023-11-09

**Authors:** César Fernández-de-las-Peñas, Ignacio Cancela-Cilleruelo, Jorge Rodríguez-Jiménez, José A. Arias-Navalón, José D. Martín-Guerrero, Oscar J. Pellicer-Valero, Lars Arendt-Nielsen, Margarita Cigarán-Méndez

**Affiliations:** ^1^Department of Physical Therapy, Occupational Therapy, Physical Medicine and Rehabilitation, Universidad Rey Juan Carlos (URJC), Madrid, Spain; ^2^Center for Neuroplasticity and Pain, Department of Health Science and Technology, School of Medicine, Aalborg University, Aalborg, Denmark; ^3^School of Medicine, Universidad CEU-San Pablo, Madrid, Spain; ^4^Intelligent Data Analysis Laboratory, Department of Electronic Engineering, ETSE (Engineering School), Universitat de València (UV), Valencia, Spain; ^5^Valencian Graduate School and Research Network of Artificial Intelligence (ValgrAI), València, Spain; ^6^Image Processing Laboratory (IPL), Universitat de València, Parc Científic, Paterna, València, Spain; ^7^Department of Gastroenterology & Hepatology, Mech-Sense, Clinical Institute, Aalborg University Hospital, Aalborg, Denmark; ^8^Steno Diabetes Center North Denmark, Clinical Institute, Aalborg University Hospital, Aalborg, Denmark; ^9^Department of Psychology, Universidad Rey Juan Carlos (URJC), Madrid, Spain

**Keywords:** COVID-19, brain fog, memory loss, concentration, Sankey plots

## Abstract

**Objective:**

This study aimed to apply Sankey plots and exponential bar plots for visualizing the trajectory of post-COVID brain fog, memory loss, and concentration loss in a cohort of previously hospitalized COVID-19 survivors.

**Methods:**

A sample of 1,266 previously hospitalized patients due to COVID-19 during the first wave of the pandemic were assessed at 8.4 (T1), 13.2 (T2), and 18.3 (T3) months after hospital discharge. They were asked about the presence of the following self-reported cognitive symptoms: brain fog (defined as self-perception of sluggish or fuzzy thinking), memory loss (defined as self-perception of unusual forgetfulness), and concentration loss (defined as self-perception of not being able to maintain attention). We asked about symptoms that individuals had not experienced previously, and they attributed them to the acute infection. Clinical and hospitalization data were collected from hospital medical records.

**Results:**

The Sankey plots revealed that the prevalence of post-COVID brain fog was 8.37% (*n* = 106) at T1, 4.7% (*n* = 60) at T2, and 5.1% (*n* = 65) at T3, whereas the prevalence of post-COVID memory loss was 14.9% (*n* = 189) at T1, 11.4% (*n* = 145) at T2, and 12.12% (*n* = 154) at T3. Finally, the prevalence of post-COVID concentration loss decreased from 6.86% (*n* = 87) at T1, to 4.78% (*n* = 60) at T2, and to 2.63% (*n* = 33) at T3. The recovery exponential curves show a decreasing trend, indicating that these post-COVID cognitive symptoms recovered in the following years after discharge. The regression models did not reveal any medical record data associated with post-COVID brain fog, memory loss, or concentration loss in the long term.

**Conclusion:**

The use of Sankey plots shows a fluctuating evolution of post-COVID brain fog, memory loss, or concentration loss during the first years after the infection. In addition, exponential bar plots revealed a decrease in the prevalence of these symptoms during the first years after hospital discharge. No risk factors were identified in this cohort.

## 1. Introduction

Although the coronavirus disease 2019 (COVID-19), a condition caused by the severe acute respiratory syndrome coronavirus 2 (SARS-CoV-2), is overall classified as a respiratory disease, there is clear evidence that it should be considered a multiorgan condition (Rabaan et al., 2023) with long-term sequelae. Neurological symptoms, e.g., ageusia, anosmia, headache, and other severe complications, e.g., delirium or stroke, are also commonly experienced in the acute phase (Kleineberg et al., 2021). Although some neurological symptoms exhibited at the acute phase of a SARS-CoV-2 infection, e.g., headache (Fernández-de-las-Peñas et al., 2021) or anosmia (Trott et al., 2022), can also be present in the post-COVID phase, other symptoms, e.g., brain fog or memory loss, are experienced *de novo* mostly after the infection (Premraj et al., 2022).

The presence of long-lasting symptoms after an acute SARS-CoV-2 infection is called long COVID (Fernández-de-las-Peñas, 2022a). A consensus Delphi study has proposed the term post-COVID-19 condition and the following definition: “Post-COVID-19 condition occurs in individuals with a history of probable or confirmed SARS-CoV-2 infection, usually 3 months from the onset of COVID-19, with symptoms that last for at least 2 months and cannot be explained by an alternative medical diagnosis. Common symptoms include, but are not limited to, fatigue, shortness of breath, and cognitive dysfunction, and generally have an impact on everyday functioning. Symptoms might be new-onset following initial recovery from an acute COVID-19 episode or persist from the initial illness. Symptoms might also fluctuate or relapse over time” (Soriano et al., 2022). Among the variety of post-COVID symptoms described in the literature, neurological symptoms, together with fatigue and pain, are among the most bothersome (Hayes et al., 2021). The presence of a post-COVID-19 condition is overall associated with a worse quality of life (Malik et al., 2022), and the presence of cognitive symptoms represents a challenge for affected individuals since these symptoms affect their daily life activities (Chen and Chen, 2020). Premraj et al. have reported prevalence rates of 32, 27, and 22% for brain fog, memory loss, and concentration loss, respectively as post-COVID symptoms 6 months later (Premraj et al., 2022). Similarly, Ceban et al. also observed a pooled prevalence of 22% for cognitive impairments during the first months following COVID-19 (Ceban et al., 2022). The Global Burden of Disease Long COVID study (1.2 million individuals with symptomatic COVID-19) found that 35.4% of COVID-19 survivors reported post-COVID cognitive symptoms during the first months after the infection (Global Burden of Disease Long COVID Collaborators et al., 2022). However, post-COVID prevalence studies are difficult to compare as different studies assess symptoms at different time points after the infection and mixed cohorts of hospitalized and non-hospitalized subjects.

Although the presence of post-COVID cognitive symptoms is associated with nervous system changes (Wu et al., 2020), it seems that these symptoms generally improve over time (Schou et al., 2021). A recent meta-analysis has identified that up to 41.7% of individuals who had surpassed COVID-19 experienced at least one post-COVID symptom 1 year after the infection and that 14.1% are unable to return to work even 2 years later (Rahmati et al., 2023). However, most studies investigating post-COVID cognitive symptomatology have used cross-sectional designs assessing the presence of these symptoms once and also had commonly used follow-up periods no longer than 1 year after the infection. The LONG-COVID-EXP study analyzed the trajectory of post-COVID cognitive symptoms, e.g., brain fog, memory loss, and concentration loss, from the onset of the infection up to the first year after hospital discharge in a cohort of individuals who were hospitalized due to COVID-19 (Fernández-de-las-Peñas et al., 2022). A better understanding of the long-term trajectory of post-COVID cognitive symptoms could have potential implications for optimizing patient interaction, treatment care, and public health outcomes (Yan et al., 2021). We present here the complete analysis of the LONG-COVID-EXP study by including data from the onset of infection, up to 6, 12, and 18 months after hospital discharge. Sankey plots and exponential bar plots are applied as a novel way to visualize the fluctuating evolution of post-COVID cognitive symptoms.

## 2. Methods

### 2.1. Participants

The LONG-COVID-EXP is a multicenter cohort study including a sample of patients who had been hospitalized by an acute SARS-CoV-2 infection confirmed at hospitalization by real-time reverse transcription-polymerase chain reaction (RT-PCR) assay of nasopharyngeal/oral swab samples and clinical symptoms during the first wave of the pandemic (March to May 2020) in five public urban hospitals in Madrid (Spain). As described, from all hospitalized patients during the first wave of the pandemic in these hospitals (*n* = 7,150), a randomly selected sample of 400 individuals from each hospital were invited to participate (Fernández-de-las-Peñas et al., 2022). The Local Ethics Committee of all the centers approved the study (HUFA20/126, HUF/EC1517, HUIL/092-20, HCSC20/495E, and HSO25112020). Verbal informed consent was obtained from all the participants before collecting data. Data from the LONG-COVID-EXP study have been previously used for identifying the trajectory of other post-COVID symptoms such as fatigue or dyspnea (Fernández-de-las-Peñas et al., 2023). In this study, we present new data on post-COVID cognitive symptoms.

### 2.2. Procedure

The procedure for this cohort study can be found elsewhere (Fernández-de-las-Peñas et al., 2022). Briefly, clinical and hospitalization data were collected from hospital medical records. Participants were scheduled for a telephonic interview conducted by healthcare professionals at 6 (T1), 12 (T2), and 18 (T3) months after hospitalization, and they were systematically asked about the presence of the following post-COVID cognitive symptoms: 1, brain fog, defined as self-perception of sluggish or fuzzy thinking; 2, memory loss, defined as self-perception of unusual forgetfulness; and/or 3, general concentration loss, defined as self-perception of not being able to maintain proper attention. We specifically asked for symptoms that subjects attributed to the SARS-CoV-2 infection and those starting no later than 3 months after their hospitalization (Soriano et al., 2022). Medical records were revised to identify if subjects self-reporting the presence of these cognitive symptoms have been diagnosed with any neurological condition explaining the symptomatology.

### 2.3. Sankey plots

Sankey plots were used as a method for visualization of the flow of quantitative data, permitting the analysis of the evolution of patients over time (Otto et al., 2022). The *x*-axis represents each timepoint assessed (6, 12, or 18 months after hospital discharge). The *y*-axis represents the percentage of individuals suffering (or not) from each particular symptom (brain fog, memory loss, and concentration loss). The darker vertical bars show the state of the subjects at that time point. The arcs depict the flows of subjects between the states (positive or negative in the symptom), with a width that is proportional to the percentage (from the total sample) of subjects in that flow. The percentage of subjects with or without each symptom is annotated on the right side of the vertical bar, whereas the flows themselves and the percentage of individuals that they contain are annotated on the left side of the vertical bar (Fernández-de-las-Peñas et al., 2023).

### 2.4. Exponential bar plots

Exponential bar plots were the method for visualization of the trajectory of the symptoms and were created with Matplotlib 3.3.4. The curve slope was fitted according to the following formula: $y=Ke^{ct}$, where y represents the modeled prevalence of the symptom (brain fog, memory loss, and concentration loss) at a time t (in months) and K and c are the parameters of the model.

### 2.5. Statistical analysis

Finally, multivariate logistic regressions, including all variables collected at hospital admission (age, sex, weight, pre-existing co-morbidities, COVID-19 onset symptoms at hospital admission, days at hospital, and ICU admission) were associated with the development of post-COVID brain fog, memory loss, and concentration loss at 12 (T2) and 18 (T3) months after using Python’s library statsmodels 0.11.1. Adjusted odds ratios (OR) with their respective confidence intervals (95% CI) were calculated. *A priori*, the level of significance was set at 0.05.

## 3. Results

From a sample of 2,000 individuals previously hospitalized due to SARS-CoV-2 during the first wave of the pandemic, a total of 1,969 (46.5% women, age: 61 years, SD: 16 years) participated at T1 (mean: 8.4, range 6 to 10); 1,593 participated at T2 (mean: 13.2, range 11 to 15); and 1,266 participated at T3 (mean: 18.3, range 16 to 21) follow-up periods. Main analyses were conducted on the total sample (*n* = 1,266, 64.3% from the initial) after completing all follow-up periods. This sample has also been included in a previous study (Fernández-de-las-Peñas et al., 2023), but the data presented in the current article are completely new and have not been previously published. Table 1 summarizes COVID-19-associated symptoms at hospital admission and medical comorbidities in the final sample (Fernández-de-las-Peñas et al., 2023).

**Table 1:** Demographic and clinical data of the sample (*n* = 1,266).

Age, mean (SD), years	61 (16.5)
Female (%)	578 (45.6%)
Weight, mean (SD), kg	74.5 (14.5)
Height, mean (SD), cm	165 (19.0)
**COVID-19-associated symptoms at hospital admission, n (%)—T0**	
Fever	948 (74.9%)
Dyspnea	361 (28.5%)
Myalgia	374 (29.5%)
Cough	360 (28.4%)
Headache	135 (16.7%)
Diarrhea	105 (8.3%)
Anosmia	105 (8.3%)
Ageusia	66 (7.0%)
Throat pain	66 (5.2%)
Vomiting	39 (3.0%)
**Medical co-morbidities**	
Hypertension	336 (26.5%)
Other (cancer, and kidney disease)	207 (16.3%)
Diabetes	158 (12.5%)
Cardiovascular disease	141 (11.2%)
Asthma	85 (6.7%)
Obesity	57 (4.5%)
Chronic obstructive pulmonary disease	47 (3.7%)
Rheumatological disease	16 (1.3%)
Stay at the hospital, mean (SD), days	10.5 (10.8)
Intensive care unit (ICU) admission	78 (6.2%)

**Figure 1:**
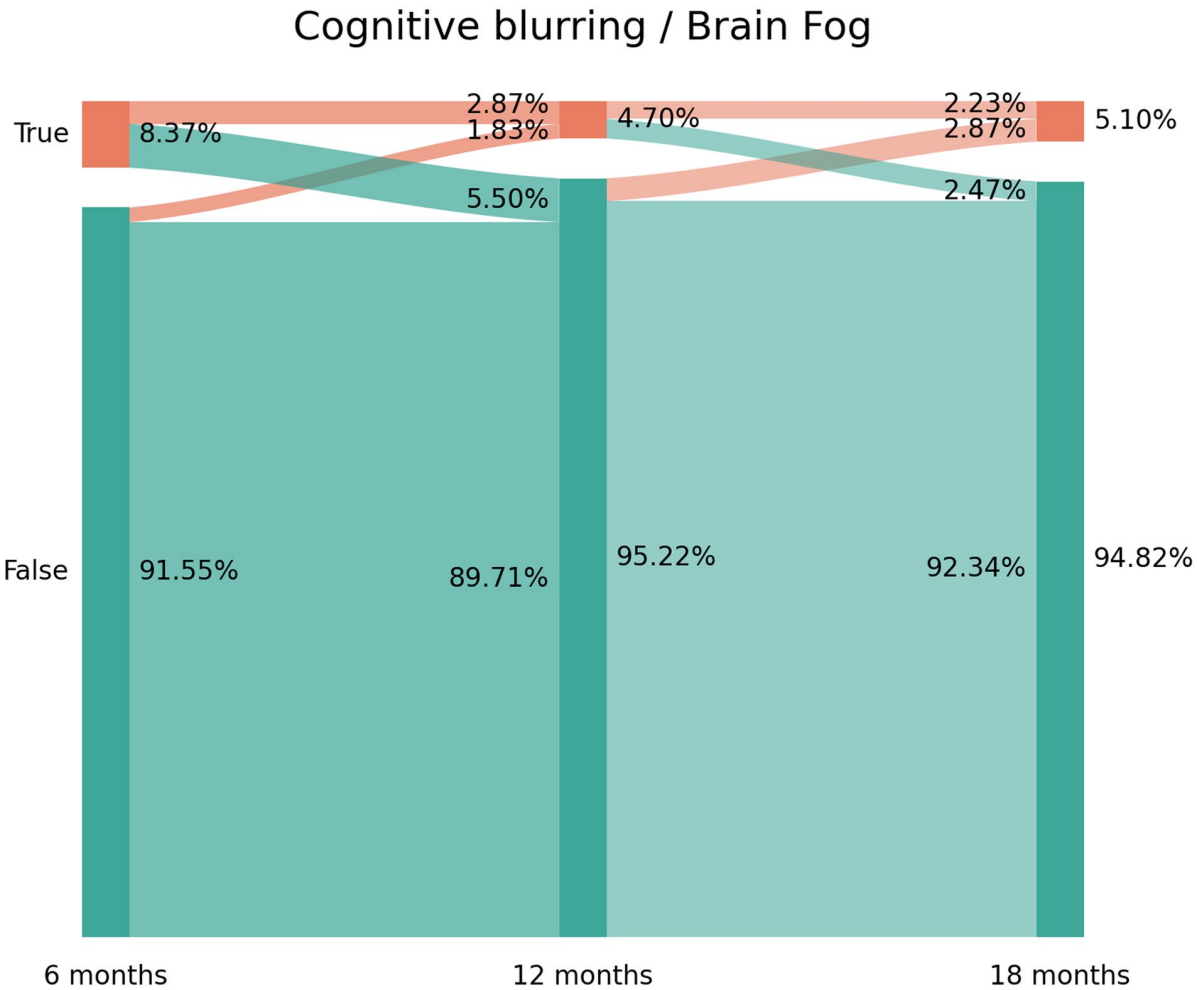
Sankey plots of brain fog during THE LONG-COVID-EXP study (from left to right): T1 (8.4 months after hospital discharge), T2 (13.2 months after hospital discharge), and T3 (18.3 months after hospital discharge).

The prevalence of post-COVID brain fog was 8.37% (*n* = 106) at T1, 4.70% (*n* = 60) at T2, and 5.10% (*n* = 65) at T3 (Figure 1). Looking at the Sankey plots of brain fog, 65% of subjects (*n* = 69/106) experiencing brain fog at T1 did not report the symptom at T2 (5.50% arc from true at T1 to false at T2). Interestingly, 38.3% (*n* = 23/60) of subjects not experiencing brain fog at T1 started to experience it at T2 (1.83% arc from false at T1 to true at T2). Overall, Sankey plots revealed that 29 patients (2.23% of the sample) exhibited post-COVID brain fog during all the follow-up periods.

**Figure 2:**
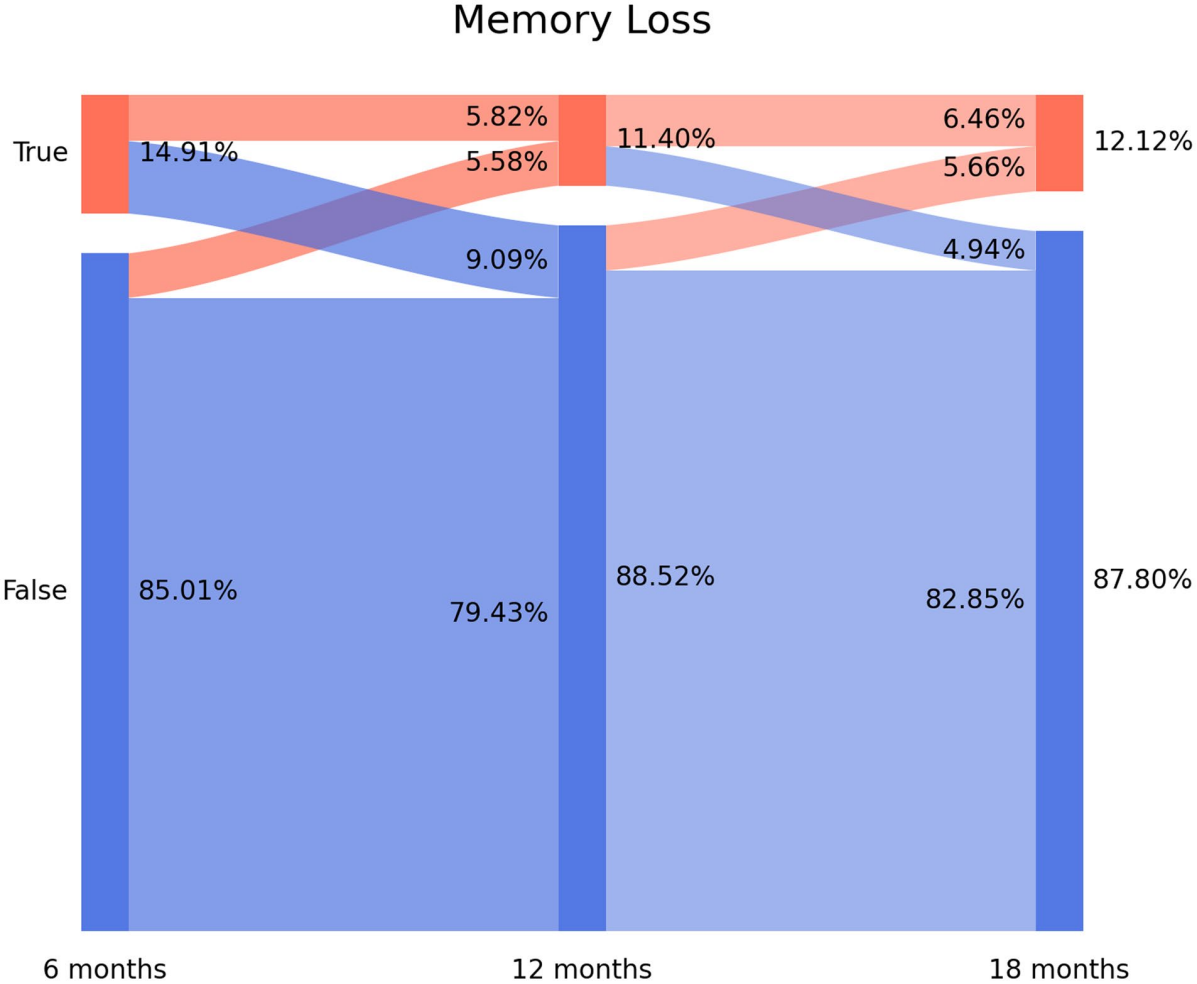
Sankey plots of memory loss during THE LONG-COVID-EXP study (from left to right): T1 (8.4 months after hospital discharge), T2 (13.2 months after hospital discharge), and T3 (18.3 months after hospital discharge).

The prevalence of post-COVID memory loss was 14.91% (*n* = 189) at T1, 11.4% (*n* = 145) at T2, and 12.12% (*n* = 154) at T3 (Figure 2). The Sankey plot showed a similar tendency to brain fog. As can be seen in Figure 2, 60.8% of the subjects (*n* = 115/189) experiencing memory loss at T1 did not report the symptom at T2 (9.09% arc from true at T1 to false at T2). Again, 47% (*n* = 73/145) of the subjects not experiencing memory loss at T1 started to report the symptom at T2 (5.58% arc from false at T1 to true at T2). The same tendency was seen between T2 and T3. Figure 2 revealed that 73 patients (5.82% of the sample) reported post-COVID memory loss during all the follow-up periods.

**Figure 3:**
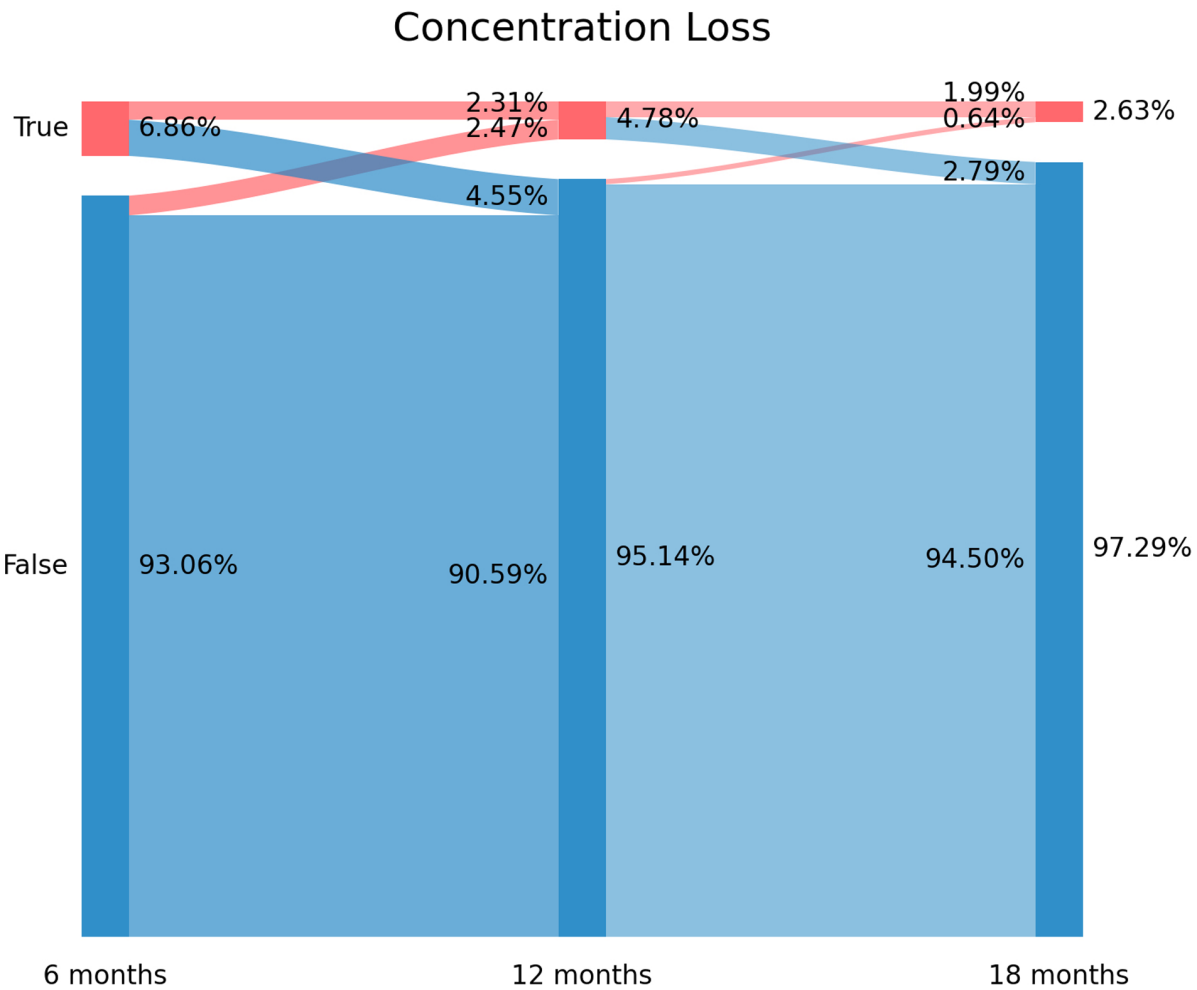
Sankey plots of concentration loss during THE LONG-COVID-EXP study (from left to right): T1 (8.4 months after hospital discharge), T2 (13.2 months after hospital discharge), and T3 (18.3 months after hospital discharge).

The prevalence of post-COVID concentration loss decreased from 6.86% (*n* = 87) at T1, to 4.78% (*n* = 60) at T2, and to 2.63% (*n* = 33) at T3. Figure 3 depicts the Sankey plots of post-COVID concentration loss and graphs showing that 65.5% of the subjects (*n* = 57/87) experiencing concentration loss at T1 did not report the symptom at T2 (4.55% arc from true at T1 to false at T2). Showing a similar tendency to brain fog and memory loss, 51.7% (*n* = 31/60) of the subjects not experiencing concentration loss at T1 experienced this post-COVID symptom at T2 (2.47% arc from false at T1 to true at T2). The Sankey plot revealed that 25 patients (1.99% of the sample) exhibited post-COVID concentration loss during all the follow-up periods.

**Figure 4:**
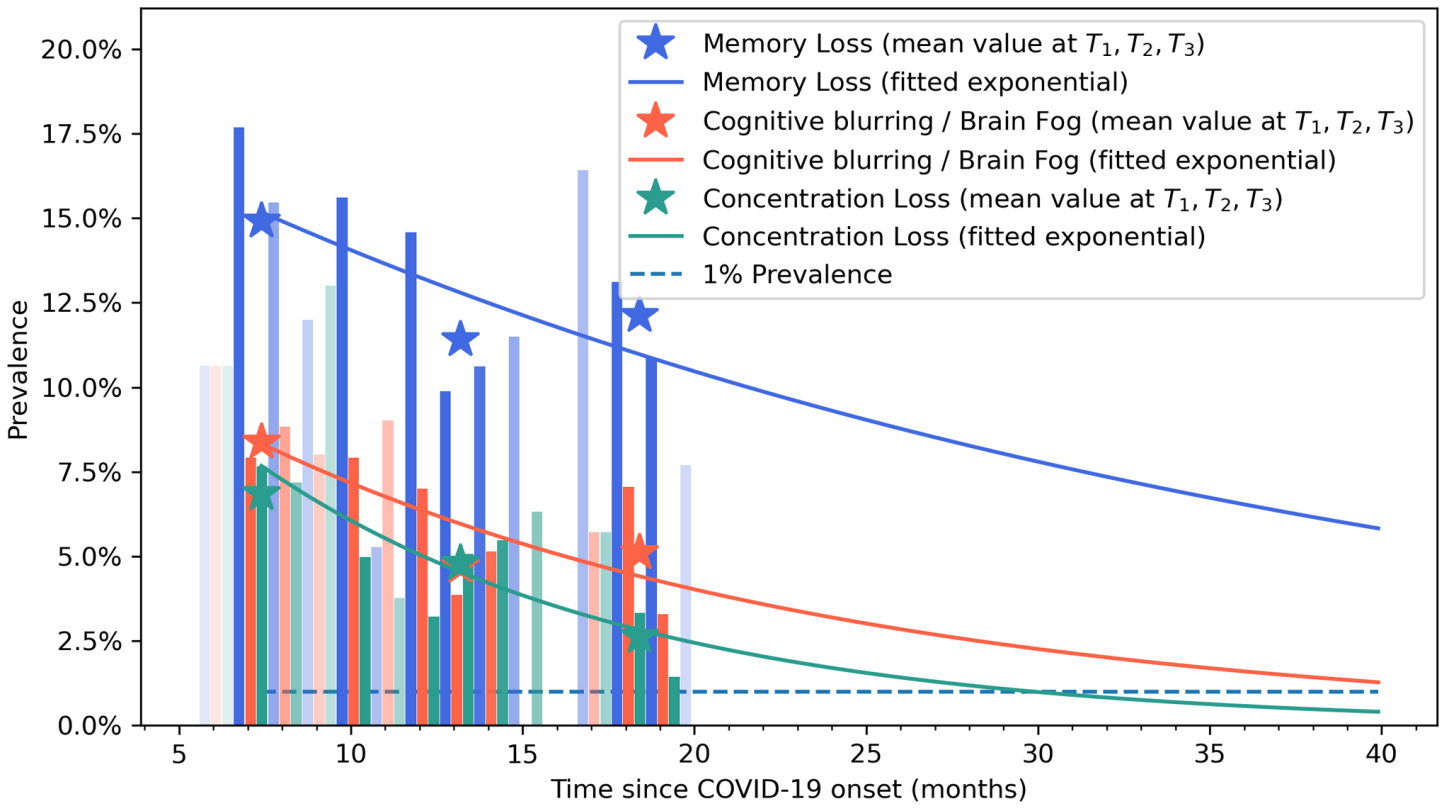
Exponential bar plots of self-reported brain fog (light orange), memory loss (blue), and concentration loss (light green) symptoms. Opacity approximately indicates the sample size at that follow-up time. Asterisks represent mean values at T0, T1, T2, and T3 follow-ups.

Figure 4 graphs the fitted exponential curves, visualizing a decreased prevalence trend in post-COVID brain fog, memory loss, and concentration loss symptoms during the years after the infection. Vertical bars represent the percentage of patients self-reporting brain fog (light orange), memory loss (blue), and concentration loss (light green). The time-point prevalence values at each post-COVID follow-up (T1, T2, and T3) are marked with asterisks in the graph.

The regression models did not reveal any data at hospitalization associated with the development of post-COVID brain fog (Table 2), memory loss (Table 3), or concentration loss (Table 4) at 12 (T2) and 18 (T3) months. The only factor associated with the development of post-COVID brain fog, memory loss, or concentration loss at T2 and T3 was experiencing the same particular symptom at the first follow-up (T1).

**Table 2:** Adjusted odds ratio (95% confidence interval) of the multivariate regression analyses of post-COVID brain fog at T2 and T3 follow-up periods.

	T2 (13.2 months)	T3 (18.3 months)
Age	1.007 (0.977, 1.039)	0.994 (0.974, 1.014)
Female sex	2.427 (0.962, 4.119)	1.058 (0.550, 2.036)
Weight	0.998 (0.971, 1.026)	1.004 (0.979, 1.030)
**Medical co-morbidities**		
Hypertension	0.669 (0.224, 1.998)	1.064 (0.535, 2.117)
Diabetes	1.592 (0.398, 4.365)	1.658 (0.736, 3.735)
Cardiovascular diseases	0.882 (0.219, 3.545)	1.027 (0.399, 2.641)
Asthma	0.453 (0.083, 2.485)	0.644 (0.710, 2.209)
Obesity	0.855 (0.100, 7.293)	1.724 (0.360, 8.250)
Chronic obstructive pulmonary disease	1.137 (0.118, 5.946)	1.202 (0.653, 2.215)
Rheumatological diseases	0.361 (0.015, 8.858)	0.789 (0.386, 1.611)
**Symptoms at hospital admission**		
Dyspnea	1.121 (0.439, 2.863)	0.961 (0.483, 1.911)
Cough	0.861 (0.348, 2.134)	1.640 (0.862, 3.120)
Myalgias	0.589 (0.224, 1.552)	0.905 (0.483, 1.991)
Headache	1.472 (0.538, 4.031)	1.117 (0.534, 2.332)
Diarrhea	1.489 (0.407, 5.442)	1.308 (0.556, 3.078)
Anosmia	1.447 (0.322, 6.504)	1.345 (0.493, 3.666)
Ageusia	0.663 (0.094, 4.288)	0.870 (0.282, 2.683)
Throat pain	2.779 (0.625, 7.354)	0.540 (0.134, 2.183)
Vomiting	0.516 (0.148, 1.793)	0.785 (0.294, 2.098)
Dizziness	1.662 (0.443, 6.244)	2.069 (0.683, 6.272)
Days at the hospital	0.950 (0.895, 1.008)	0.979 (0.947, 1.011)
Intensive care unit (ICU) admission	0.863 (0.316, 2.355)	0.966 (0.374, 2.490)
Brain fog at T1 (8.4 months)	6.867 (3.477, 13.563)^#^	7.290 (3.773, 14.087)^#^

^#^*p* < 0.001.

**Table 3:** Adjusted odds ratio (95% confidence interval) of the multivariate regression analyses of post-COVID memory loss at T2 and T3 follow-up periods.

	T2 (13.2 months)	T3 (18.3 months)
Age	1.010 (0.993, 1.026)	0.987 (0.958, 1.016)
Female sex	0.951 (0.597, 1.515)	1.000 (0.633, 1.578)
Weight	1.008 (0.977, 1.039)	0.992 (0.976, 1.008)
**Medical co-morbidities**		
Hypertension	1.309 (0.821, 2.089)	1.100 (0.697, 1.578)
Diabetes	0.973 (0.531, 1.784)	0.690 (0.368, 1.293)
Cardiovascular diseases	1.105 (0.605, 2.016)	1.083 (0.607, 1.935)
Asthma	0.776 (0.368, 1.638)	0.664 (0.313, 1.409)
Obesity	1.849 (0.656, 5.210)	1.048 (0.573, 1.919)
Chronic obstructive pulmonary disease	1.067 (0.376, 3.030)	0.573 (0.185, 1.771)
Rheumatological diseases	0.975 (0.193, 4.934)	1.044 (0.627, 1.737)
**Symptoms at hospital admission**		
Dyspnea	1.003 (0.628, 1.602)	1.193 (0.755, 1.884)
Cough	1.238 (0.777, 1.972)	1.187 (0.775, 1.865)
Myalgias	0.937 (0.588, 1.494)	0.898 (0.566, 1.427)
Headache	1.255 (0.742, 2.122)	0.908 (0.516, 1.598)
Diarrhea	1.013 (0.537, 1.911)	1.373 (0.736, 2.561)
Anosmia	0.846 (0.381, 1.879)	0.871 (0.382, 1.986)
Ageusia	1.754 (0.836, 3.677)	1.233 (0.589, 2.584)
Throat pain	1.313 (0.576, 2.993)	1.733 (0.793, 3.788)
Vomiting	1.309 (0.449, 3.816)	1.563 (0.583, 4.188)
Dizziness	0.833 (0.320, 2.170)	1.957 (0.841, 4.554)
Days at the hospital	0.959 (0.522, 1.176)	0.868 (0.462, 1.631)
Intensive care unit (ICU) admission	0.825 (0.441, 1.540)	0.889 (0.484, 1.630)
Memory loss at T1 (8.4 months)	8.054 (5.196, 12.484)^#^	6.317 (4.109, 9.713)^#^

^#^*p* < 0.001.

**Table 4:** Adjusted odds ratio (95% confidence interval) of the multivariate regression analyses of post-COVID concentration loss at T2 and T3 follow-up periods.

	T2 (13.2 months)	T3 (18.3 months)
Age	1.012 (0.986, 1.039)	0.998 (0.976, 1.020)
Female sex	1.763 (0.814, 3.820)	1.340 (0.619, 2.903)
Weight	1.008 (0.986, 1.032)	1.004 (0.979, 1.030)
**Medical co-morbidities**		
Hypertension	1.202 (0.568, 2.542)	0.642 (0.628, 1.428)
Diabetes	1.641 (0.663, 4.062)	0.450 (0.124, 1.635)
Cardiovascular diseases	1.808 (0.731, 4.471)	0.764 (0.233, 2.511)
Asthma	1.172 (0.372, 3.693)	0.520 (0.134, 2.007)
Obesity	1.017 (0.465, 2.223)	2.100 (0.468, 9.431)
Chronic obstructive pulmonary disease	1.808 (0.443, 7.383)	1.284 (0.236, 3.987)
Rheumatological diseases	1.263 (0.519, 3.073)	1.017 (0.104, 6.933)
**Symptoms at hospital admission**		
Dyspnea	1.149 (0.538, 2.455)	0.774 (0.345, 1.602)
Cough	0.589 (0.269, 1.292)	0.476 (0.210, 1.080)
Myalgias	1.038 (0.508, 2.124)	0.789 (0.386, 1.611)
Headache	0.688 (0.263, 1.802)	1.402 (0.636, 3.095)
Diarrhea	0.818 (0.303, 2.206)	0.908 (0.346, 2.381)
Anosmia	1.447 (0.322, 6.504)	1.304 (0.387, 4.395)
Ageusia	0.265 (0.050, 1.400)	1.352 (0.267, 5.846)
Throat pain	0.414 (0.077, 2.231)	0.678 (0.172, 2.675)
Vomiting	1.947 (0.373, 7.150)	1.813 (0.492, 5.686)
Dizziness	1.190 (0.325, 4.352)	0.850 (0.200, 3.618)
Days at the hospital	0.985 (0.950, 1.020)	0.988 (0.957, 1.020)
Intensive care unit (ICU) admission	0.559 (0.179, 1.748)	1.343 (0.475, 3.798)
Concentration loss at T1 (8.4 months)	19.467 (9.390, 40.357)^#^	5.686 (3.154, 10.252)^#^

^#^*p* < 0.001 and ^*^p < 0.05.

## 4. Discussion

This is the first post-COVID study using Sankey plots and exponential bar curves as two visualization approaches for assessing the recovery trajectory of post-COVID cognitive symptoms in individuals who had been previously hospitalized due to SARS-CoV-2. The Sankey plots revealed a fluctuating nature of post-COVID brain fog, memory loss, and concentration loss during the first year after COVID-19. Thus, exponential bar plots revealed a progressive decrease in the prevalence of post-COVID cognitive symptomatology during the first years after the infection.

Previous meta-analyses, including cross-sectional studies, reported an overall prevalence of post-COVID cognitive impairments ranging from 22 to 35% during the first 6 months after infection (Ceban et al., 2022; Global Burden of Disease Long COVID Collaborators et al., 2022). O’Mahoney et al. reported an overall prevalence of cognitive impairment ranging from 17 to 20% in hospitalized COVID-19 survivors 6 months after infection (O’Mahoney et al., 2022). Both meta-analyses did not differentiate between particular cognitive impairments; accordingly, the prevalence rate cannot be compared with our study. Premraj et al. provided prevalence rates for brain fog, memory loss, and concentration loss separately ranging from 22 to 32% 6 months after the infection (Premraj et al., 2022). The current study showed prevalent rates of post-COVID cognitive symptoms lower than in the former literature (Ceban et al., 2022; Global Burden of Disease Long COVID Collaborators et al., 2022; Premraj et al., 2022). Differences in designs (cross-sectional vs. longitudinal), follow-ups (6–12 months after), population (hospitalized vs. non-hospitalized COVID-19 survivors), and collection procedures (self-reported, phone, and face-to-face) may explain the heterogeneous prevalence rates among studies. Additionally, the age of the sample can also influence the presence of cognitive impairments, and we should consider that the age of our sample was older than 60. Furthermore, since cognitive problems include heterogeneous symptomatology, it is possible that some individuals are not able to distinguish between specific symptoms.

The use of Sankey plots has permitted us to visualize the fluctuating nature of post-COVID cognitive symptomatology, as previously suggested (Fernández-de-las-Peñas, 2022b):

1. New-onset post-COVID cognitive symptom: subjects experiencing brain fog (8.37% true vertical bar at T1 on Figure 1), memory loss (14.91% true vertical bar at T1 on Figure 2), or concentration loss (6.86% true vertical bar at T1 on Figure 3) after the infection, and they did not experience this symptom before the infection;
2. Delayed post-COVID cognitive symptom: subjects reporting post-COVID brain fog (1.83% arc from false at T1 to true at T2 in Figure 1), memory loss (5.58% from false at T1 to true at T2 in Figure 2), or concentration loss (2.47% from false at T1 to true at T2 in Figure 3) at a longer follow-up period, i.e., with a delayed in time, in relation to the acute phase of the infection;
3. Persistent post-COVID cognitive symptom: individuals suffering from post-COVID brain fog (2.23% of the sample in Figure 1), memory loss (5.82% of the sample in Figure 2), or concentration loss (1.99% of the sample in Figure 3) throughout the entire follow-up period.

The terms “new-onset,” “delayed-onset,” and “persistent” post-COVID symptoms were previously proposed by Fernández-de-las-Peñas et al. (2021). The use of the Sankey plot has permitted the identification of these symptoms in a cohort of hospitalized patients. By definition, a new-onset and persistent post-COVID symptom can be easily attributable to the SARS-CoV-2 infection if the symptom starts no later than 3 months after COVID-19 (Soriano et al., 2022). The “delayed-onset” post-COVID symptom is more difficult to attribute to COVID-19 since it appears months later. This finding would support the hypothesis that COVID-19 might trigger a latent neurodegenerative process with residual damage, persistent immune activation, or the unmasking of underlying co-morbidities (Korompoki et al., 2021). Other associated factors (e.g., post-traumatic stress disorder, medical comorbidities, reinfections, and increasing age) may also be related to the development of “delayed” post-COVID cognitive symptoms.

Post-COVID cognitive symptoms may arise from a combination of biological factors, e.g., persistent viral damage, neuroinflammation, damage to the blood–brain barrier, neural network dysfunction, or altered excitability and neurotransmission in the primary motor cortex (Ortelli et al., 2002; Burks et al., 2021; Churchill et al., 2023), as well as psychological factors, e.g., anxiety, depression, or post-traumatic stress disorder (PTSD) (Liu et al., 2023). Considering the long regeneration time of nervous system neurons, the recovery of post-COVID cognitive symptoms could be longer than expected. The exponential bar plots visualized that the prevalence of post-COVID brain fog, memory loss, and concentration loss can last up to 5 years after acute infection. The bar plots showed a higher prevalence of memory loss when compared with concentration loss or brain fog. Accordingly, the recovery curve of memory loss indicates that this post-COVID cognitive symptom will persist for a longer period of time. Recent evidence reported that transcriptomic alterations within the central nervous system, long-lasting activation of the immune cells, and impaired hippocampal neurogenesis have a role in the neurological manifestations observed in animal models infected with SARS-CoV-2 (Usai et al., 2023); however, no single mechanism explains all post-COVID cognitive symptoms seen in humans (Ali Awan et al., 2021).

The presence of post-COVID cognitive symptomatology represents a challenge for individuals with long COVID since these symptoms affect their daily life activities (Malik et al., 2022). In addition, early self-perception of cognitive deficits in the first month after an acute SARS-CoV-2 infection is associated with suffering from long COVID symptoms (Liu et al., 2023). Hence, early identification of risk factors associated with this symptomatology could help improve their management. Ceban et al. found that female sex, older age, and internal care unit (ICU) admission were factors associated with post-COVID cognitive symptomatology (Ceban et al., 2022). That female sex is a risk factor associated with post-COVID symptoms is supported by current literature (Tsampasian et al., 2023). We did not find this association between female sex and post-COVID brain fog, memory loss, or concentration loss in our cohort of hospitalized COVID-19 survivors. Similarly, no effect of age was found. This lack of effect could be associated with the fact that the average age of our sample was 61 years, and the analyses were not able to identify the effect of age. Cognitive fragility is well associated with older age, and the prevalence of post-COVID cognitive symptoms could be higher in an older population; however, we believe that this effect would not affect the fluctuating nature and evolution of post-COVID cognitive symptomatology seen with Sankey and exponential bar plots. Thus, multivariate analyses did not find any significant factor associated with the development of long-term post-COVID cognitive symptomatology in our sample of previously hospitalized COVID-19 survivors. It is possible that other risk factors not included in this study, e.g., differences in neurodegenerative or neuroinflammation biomarkers or psychological aspects, could be associated with post-COVID cognitive symptomatology. Furthermore, our study focused on self-reported post-COVID cognitive symptoms; however, there is evidence suggesting that executive function is also affected in people hospitalized by COVID-19 and with long COVID symptoms (Ariza et al., 2023).

Although the current study used two novel methods for visualizing post-COVID cognitive symptomatology, the results should be taken into consideration after looking at potential limitations. First, the current cohort just included previously hospitalized COVID-19 survivors. We do not know if non-hospitalized COVID-19 survivors will exhibit similar results. Second, we collected self-reported symptomatology by telephonic interview, which could have a potential bias. However, the use of telephonic interviews is the only way to assess large cohorts (over 1,000 patients during long-term follow-up periods). In addition, the fact that cognitive symptoms were self-reported could lead to an underestimation of these symptoms if they had been assessed objectively. Finally, psychological factors such as anxiety, depression, or PTSD were not included. Since PTSD is present in almost 14.6% of subjects with long COVID 1 year after infection (Yang et al., 2022), future studies should include these variables.

## 5. Conclusion

This study reveals, by using the Sankey plot, a fluctuating evolution of self-reported post-COVID brain fog, memory loss, and concentration loss symptoms during the first year after an acute SARS-CoV-2 infection in previously hospitalized COVID-19 survivors. The use of exponential bar plots showed a decrease in the prevalence of these symptoms in the first 3–4 years after hospitalization. No associated risk factors were identified.

## Data availability statement

The original contributions presented in the study are included in the article/supplementary material, further inquiries can be directed to the corresponding author.

## Ethics statement

The studies involving humans were approved by The Local Ethics Committee of all centers approved the study (HUFA20/126, HUF/EC1517, HUIL/092-20, HCSC20/495E, HSO25112020). The studies were conducted in accordance with the local legislation and institutional requirements. The participants provided their written informed consent to participate in this study.

## Author contributions

CF-d-l-P: Conceptualization, Data curation, Funding acquisition, Investigation, Methodology, Validation, Visualization, Writing – original draft, Writing – review & editing. IC-C: Conceptualization, Data curation, Investigation, Methodology, Validation, Visualization, Writing – original draft, Writing – review & editing. JR-J: Conceptualization, Data curation, Investigation, Methodology, Validation, Visualization, Writing – original draft, Writing – review & editing. JA-N: Conceptualization, Data curation, Investigation, Methodology, Project administration, Resources, Software, Supervision, Validation, Visualization, Writing – review & editing. JM-G: Data curation, Formal analysis, Investigation, Methodology, Project administration, Resources, Software, Supervision, Validation, Visualization, Writing – review & editing. OP-V: Data curation, Formal analysis, Investigation, Project administration, Resources, Software, Supervision, Writing – review & editing. LA-N: Conceptualization, Investigation, Methodology, Project administration, Resources, Supervision, Validation, Visualization, Writing – original draft, Writing – review & editing. MC-M: Conceptualization, Data curation, Investigation, Methodology, Project administration, Resources, Supervision, Validation, Visualization, Writing – review & editing.
